# Automated Micro-Crack Detection within Photovoltaic Manufacturing Facility via Ground Modelling for a Regularized Convolutional Network

**DOI:** 10.3390/s23136235

**Published:** 2023-07-07

**Authors:** Damilola Animashaun, Muhammad Hussain

**Affiliations:** Department of Computer Science, Centre for Industrial Analytics, School of Computing and Engineering, University of Huddersfield, Queensgate, Huddersfield HD1 3DH, UK; u2260788@unimail.hud.ac.uk

**Keywords:** defect detection, micro-cracks, photovoltaics, smart manufacturing, quality inspection

## Abstract

The manufacturing of photovoltaic cells is a complex and intensive process involving the exposure of the cell surface to high temperature differentials and external pressure, which can lead to the development of surface defects, such as micro-cracks. Currently, domain experts manually inspect the cell surface to detect micro-cracks, a process that is subject to human bias, high error rates, fatigue, and labor costs. To overcome the need for domain experts, this research proposes modelling cell surfaces via representative augmentations grounded in production floor conditions. The modelled dataset is then used as input for a custom ‘lightweight’ convolutional neural network architecture for training a robust, noninvasive classifier, essentially presenting an automated micro-crack detector. In addition to data modelling, the proposed architecture is further regularized using several regularization strategies to enhance performance, achieving an overall F1-score of 85%.

## 1. Introduction

The issue of global emissions and how to address them is a globally shared concern, leading to the emergence of the renewable energy field, and among the practical options available at all levels of society, solar power is the most widely accepted [1]. According to the International Energy Agency (IEA), global carbon dioxide (CO_2_) emissions from energy combustion and industrial processes increased by 0.9% to a record high of 36.8 Gt in 2022 after two years of pandemic-related oscillations, with CO_2_ emissions from energy combustion rising by 1.3% in 2022 while CO_2_ emissions from industrial processes declined [2]. 

The use of solar energy has resulted in more photovoltaic (PV) solar panels being produced, installed, and maintained. It is crucial to have a dependable inspection process as production is automated to meet demand. These panels may face challenges, like soiling, harsh environments, and damage, which can lower their performance [1,3,4,5]. These defects may be in the form of micro-cracks, which can be hard to visually identify [6], and their manual detection is subject to human error and thus susceptible to low efficiency, high labor costs, high rates of false detection, as well as a high scrap rate [7]; hence, there is a need to develop an automated process for easy detection.

This study explains how the manual inspection of PV cells in manufacturing facilities is a costly and time-consuming process that can result in human bias. The solution to this problem is integrating computer vision into the inspection process, which can detect defective PV cells more quickly and cost effectively. Data collection from within manufacturing facilities can be a cumbersome task due to several issues, including limited accessibility and down-time in the event of needing to deploy an acquisition mechanism for data collection. The complex and sensitive nature of PV manufacturing means researchers cannot simply collect data from a PV manufacturing site; hence, this work proposes the modeling of production floor variance in order to scale a small PV dataset in a representative manner, followed by the development of a lightweight CNN architecture for the on-site, automated detection of micro-cracks occurring during the manufacturing process.

### 1.1. Literature Review

The popularity and affordability of solar power have led to increased use of translucent solar panels in homes and businesses. However, in utility-scale solar power plants, defects in photovoltaic modules, such as micro-cracks, must be identified to maintain efficiency. Gabor et al. [8] examined the potential of UV fluorescence (UVF) for detecting cracked cells in solar panels via a pole-mounted UV flash camera system applied to residential rooftops in Boulder, Colorado, and they found that the pole-mounted UVF system is highly applicable and informative for detecting defects for a range of residential panel ages and designs, and it can provide additional information to that from electroluminescence imaging. Han et al. [9] proposed a deep learning approach using an improved version of YOLOv3-tiny to detect faults in solar panels with the aid of a UAV equipped with a thermal camera and GPS to acquire thermal images and locate faults. The information is transmitted to a remote server for visualization via long-term evolution (LTE), and the proposed DL model outperforms the current default YOLOv3-tiny model, achieving a high accuracy of 96.45%.

Espinosa et al. [10] proposed using a CNN to automatically classify physical faults in PV plants by segmenting and classifying RGB images, and they included experimental results for both two output classes (no fault and fault) and four output classes (no fault, dust, cracks, and shadows), achieving an average accuracy of 75% for the two output classes and 70% for the four output classes, which demonstrated its potential as a classification method for PV systems. Acharya et al. [11] also proposed a method for classifying different types of defects in solar cells using a deep Siamese convolutional neural network (CNN). The EL image is first preprocessed to remove noise and distortions, and then the proposed model is tested on a standard EL image dataset. Simulation results show that the proposed model achieves better classification accuracy with a 90% AUC in detecting defective solar cells. 

While using advanced CNN architectures and ensemble learning to detect micro-cracks in EL images of PV modules, Rahman et al. [12] achieved high accuracy rates of 97.06% and 96.97% for polycrystalline and monocrystalline solar panels, respectively, by utilizing pre-trained models, including Inception-v3, VGG-19, VGG-16, Inception-ResNet50-v2, Xception, and ResNet50-v2 [13]. Akram et al. [14], on the other hand, adopted a CNN-based deep learning architecture using an “isolated model” which had been trained with samples from the EL PV cell and employed transfer learning for fine-tuning the architecture, achieving an accuracy rate of 99.23%, though the generalization and accurate representativeness of the trained model may raise concerns due to the size of the dataset. However, Mathias et al. [15] expanded the study by training 2000 EL images and testing 300 EL images. The preprocessing stage involved applying perspective transformation and separating the solar panel section and individual solar cells from the PV panel. Textural features were extracted from these cells using DWT and SWT. Support vector machine and back propagation neural network were used for classification into cracked and non-cracked cells, and the researchers achieved high classification accuracies of 92.67% and 93.67% using SVM and BPNN, respectively. Winston et al. [16] also adopted this model, using six input parameters, and both methods showed promising results with average accuracies of 87% and 99%, respectively, and an F1-score of 94.6%, recall of 96.3%, and precision of 87.3% [17].

In the study of Xue et al. [18], the authors adopted fuzzy c-means clustering and AlexNet CNN [4] to accurately detect hidden cracks despite an irregular and composite texture background, thereby achieving stable and precise results with 94.4% accuracy [19].

In summary, current research on automating the detection of faults in PV systems lacks practical considerations. Although several works have focused on optimizing state-of-the-art CNN architectures for high accuracy, there has been little attention on developing lightweight CNN architecture, i.e., internal architectural complexity. This is a key area for focus as the majority of the state-of-the-art architectures cannot be deployed onto constrained-edge devices due to the high computational complexity of the internal network. Hence, production sites would need to commission high-performance computing, i.e., GPUs, to run state-of-the art CNNs, such as VGG, which significantly increases the cost.

### 1.2. Paper Contribution

This study has two fundamental contributions. Firstly, as evident from the literature review, the collection of quality PV cell samples for normal and defective cell surfaces is a key component when looking to develop automated CNN algorithms for defect detection and classification. However, the procurement of quality datasets, in particular EL-processed samples can be cumbersome and sometimes practically infeasible due to access restrictions within certain manufacturing facilities. Hence, to provide an alternative route, we present the modelling of internal and external variance in the context of PV cell manufacturing conditions by proposing representative augmentations for appropriately scaling and increasing the variance of EL-based PV datasets. Secondly, a custom CNN architecture with a lightweight footprint is developed (4.67 Million parameters) and trained using the augmented-generated samples. The design and training of a ‘lightweight’ architecture is to address the stringent deployment conditions within manufacturing facilities, such as edge-device deployment, low power consumption, and close-to-the-source inferencing, in addition to generalization via scaled augmentations several regularization techniques, which are applied for further model generalization and to reduce the degree of overfitting.

## 2. Methodology

### 2.1. Dataset

For the purpose of this study, a dataset of PV-cell images from the manufacturing facility was used and was manually labeled by experts. 

The dataset has two classes, normal and defective, with a small sample size of 930, which makes it difficult to develop a highly generalized architecture capable of accurately distinguishing between the two classes. Table 1 presents the status of the dataset.

**Figure 1:**
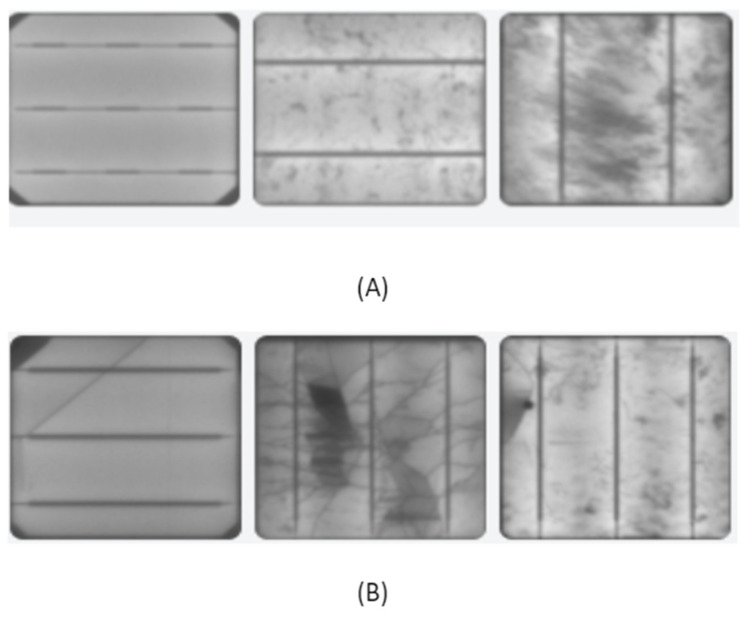
Data investigation: (**A**) normal, (**B**) defective.

Figure 1 shows examples of normal and defective PV-cell surfaces. To ensure proper scaling of the dataset, it was necessary to understand the visual differentiation features and variance of the two classes. By observing Figure 1A, we can identify differences in texture and global-level variance. For instance, the normal class has texture variance, with the first image being clearer than the center image and the center image being clearer than the last image. It’s crucial to consider this variance as it may result in the developed architecture falsely generalizing that only clear surface images belong to the normal class.

In Figure 1, the visual differences between the normal and defective PV-cell surfaces are impacted by both internal and external factors. For example, shading or poor filter quality can induce pixel shading on normal cells, which can resemble micro-cracks on defective cells and increase the chance of misclassification. 

The busbar is a crucial component of PV cells, but its configuration and starkness can vary significantly, potentially leading to misclassification. Therefore, these observations suggest that the developed architecture needs to account for various degrees of textural and internal variance within and between classes to achieve accurate classification.

Despite the small and representative size of the original dataset, it was spliced using the train, test, and split function (in the ratio 70:10:20) as shown in Table 2.

### 2.2. Data Augmentations

Upon analyzing the dataset, it was hypothesized that addressing the variance within the dataset could be achieved through representative data modeling, rather than randomly augmenting the dataset to increase its size. Consequently, the dataset was augmented and limited to 2232 samples from the initial 930 images. This decision was also influenced by practical limitations in obtaining PV data from manufacturing facilities due to limited access and a lack of open-source data.

Data transformations can be divided into two categories: translational invariance and translational equivariance. The former is used for designing the internal layers of architectures and preserves regional transformations through aggregation and is represented as the vector sum of a constant v to every point x, as expressed in (1) and (2):

(1) $$T_{v}\;\left(x\right)\;=\;x\;+v$$

(2) $$f\left(g\left(x\right)\right)\;=\;g\left(f\left(x\right)\right)$$

The latter, on the other hand, is used for data scaling and transforms (g) the input image (f) according to the type of transformation applied. This is mathematically expressed as follows:

The selection of augmentations was based on generating representative samples that may be generated in PV manufacturing facilities, considering different factors, such as production line configurations and EL camera specifications.

#### 2.2.1. Scaling Variability

**Figure 2:**
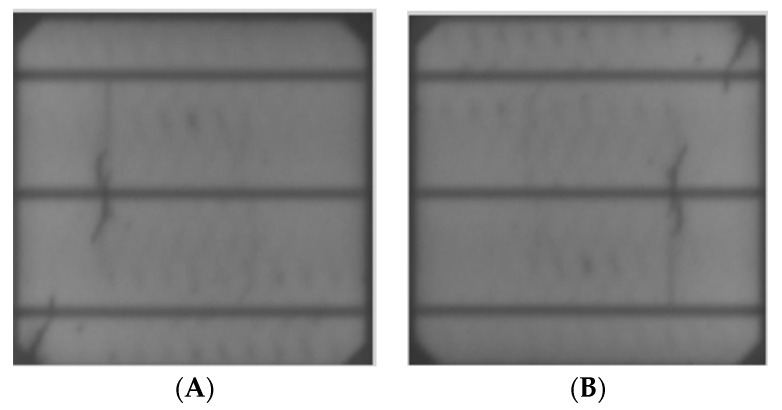
Flip: (**A**) vertical, (**B**) horizontal.

**Figure 3:**
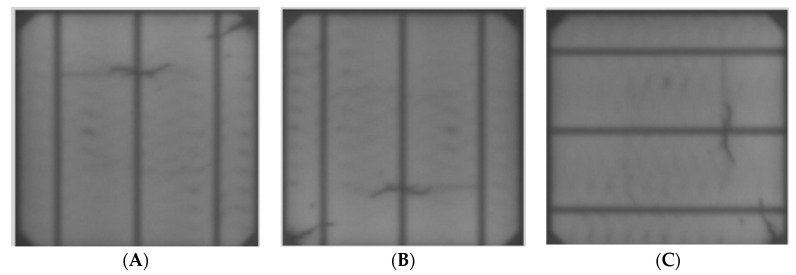
Rotation: (**A**) clockwise, (**B**) counter-clockwise, (**C**) 180 degrees shift.

**Figure 4:**
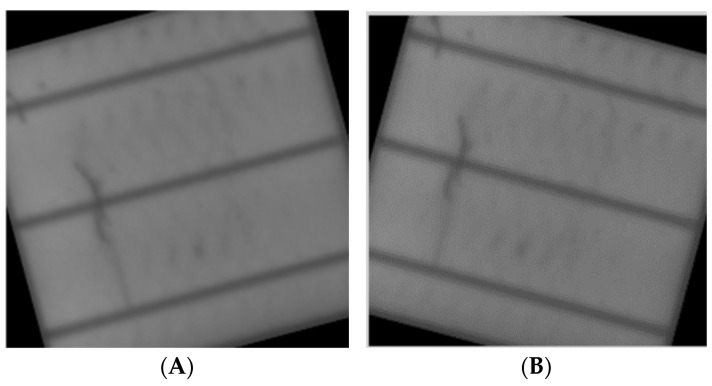
15 Degree: (**A**)vertical shift, (**B**) horizontal shift.

**Figure 5:**
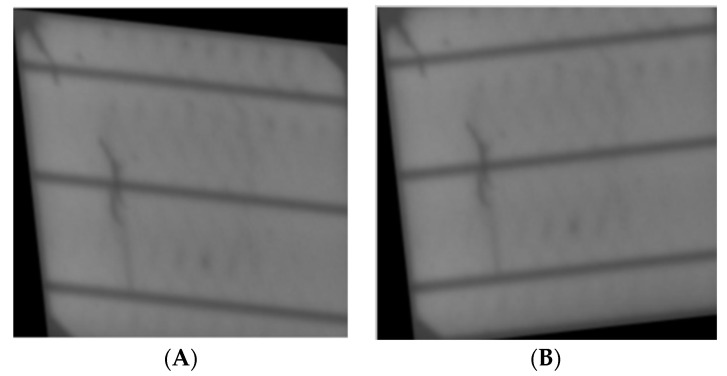
15 Degree Rotation: (**A**) height shift, (**B**) width shift.

The production of a PV cell goes through various stages from silicon ingots down to cell assembly, all of which involves various texturing processes and quality control at all stages. These variabilities may result in different cell surface orientation. Therefore, to ensure consistency and enable the proposed model to detect the different instances, various scaling variabilities, like flipping, rotation, weight shift, and height shift, were applied, as shown in Figure 2, Figure 3, Figure 4 and Figure 5.

#### 2.2.2. Contrast Variability

**Figure 6:**
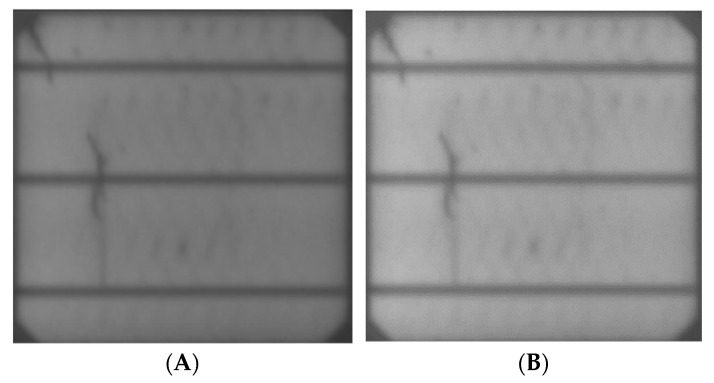
Brightness: (**A**) input, (**B**) output.

**Figure 7:**
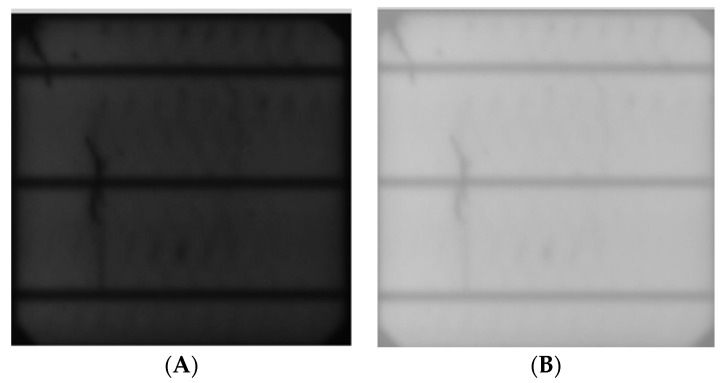
Exposure: (**A**) input, (**B**) output.

**Figure 8:**
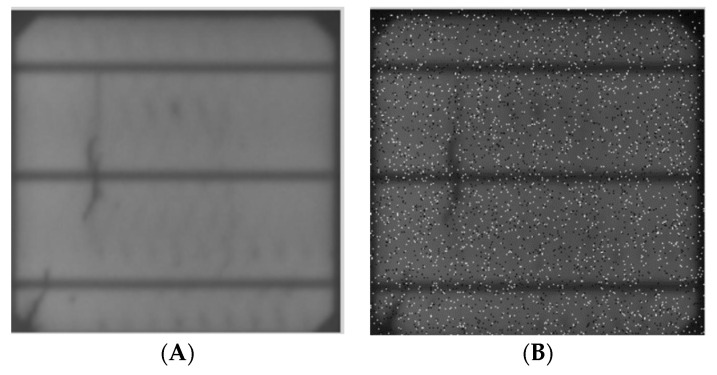
Noise: (**A**) input, (**B**) output.

Different samples of the PV cells were taken under different environmental factors, like the dimness of the room when the pictures were taken, camera quality, or dust, which could have accumulated from the rigorous production and inspection stages, hence the need to ensure the model is able to understand these variations by adding contrast augmentations, like brightness, exposure, and noise, to the dataset, as shown in Figure 6, Figure 7 and Figure 8. 

Table 3 presents the state of the newly generated dataset after applying the aforementioned augmentation techniques. The dataset was then split, as shown in Table 4, using the same ratio as the original dataset.

### 2.3. Proposed Architecture

To reduce the complexity of the automated defect detector, a custom CNN architecture was developed featuring two convolutional blocks with a limited number of filters. Filters are an important component within a CNN architecture, as they aim to extract key features, but at the same time, a high number of filters can increase the computational cost. Hence, our strategy was based on applying representative augmentations to accentuate key features and variance within the dataset, to make it easier for filters to grasp the underlying characteristics, using only a limited number of filters.

Each convolutional block is comprised of predefined filters followed by feature aggregation via Max-pooling, with the result feeding into the ReLu activation function. The selection of ReLu was again in line with our research theme, i.e., lightweight footprint, as it simply implied a Max operation, as expressed in (3).

(3) $$s\left(x\right)=\max(0,x)$$

The first convolutional block contained eight filters, also known as kernels. The feature map details that the operations performed in the first convolutional block were required as input data for the second convolutional block. We decided to start with a small number of filters, then incrementally increase the quantity, if required. The rationale for this was that filters contribute to increased computational parameters, and as our aim was to produce a computationally lightweight architecture, opting for a large number of filters would be counterproductive towards this aim.

**Figure 9:**
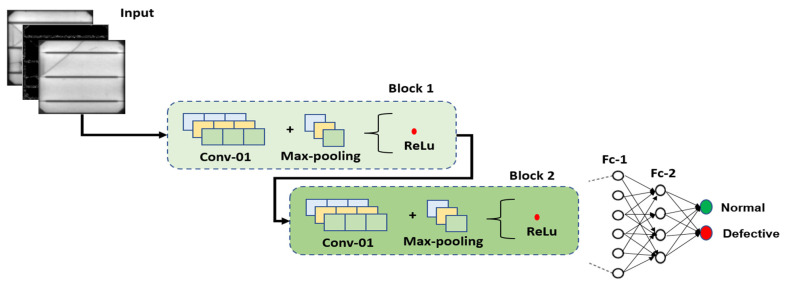
Proposed architecture.

Figure 9 presents the proposed architecture containing two convolutional blocks followed by two fully connected layers feeding into the output. As presented in Figure 9, each convolution block contained a predefined number of filters followed by feature map aggregation and non-linearity transformation via the ReLu activation function.

Table 5 presents the internal architectural depth details for the proposed architecture. As evident from Table 5, the proposed architecture resulted in only 4.67 million parameters. This would be considered lightweight compared to other architectures, such as ResNet at 11.69 Million [20] and VGG with over 100 Million parameters [21].

## 3. Model Evaluation

### 3.1. Hyperparameter Tuning

This section compares the performance of the various experimental processes to ascertain the optimal architecture configuration using Google Colab for GPU acceleration. Due to limited GPU access, training was capped at 50 epochs, batch size was set to 32, learning rate was set to 0.02, and SGD-M optimizer was adopted for faster training, as shown in Table 6.

### 3.2. Original Dataset Performance

**Figure 10:**
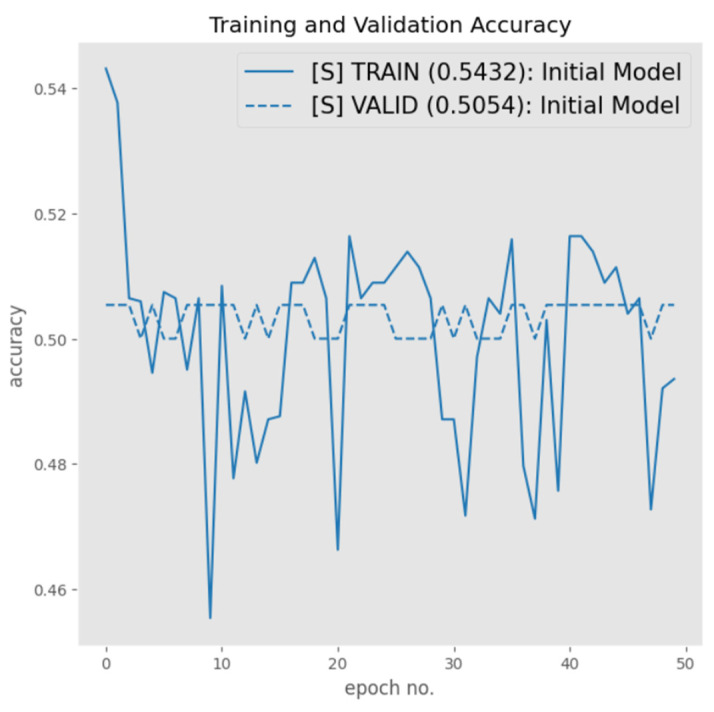
Original data performance.

Following the data split in Table 2, the proposed architecture was explored using a learning rate of 0.02 for a fair comparison, and the results are shown in Figure 10, which represents the model performance. It is evident from Figure 10 that the initial model was not able to provide satisfactory results with a validation accuracy of 50.54%, i.e., the architecture was essentially rendered as a random classifier.

**Figure 11:**
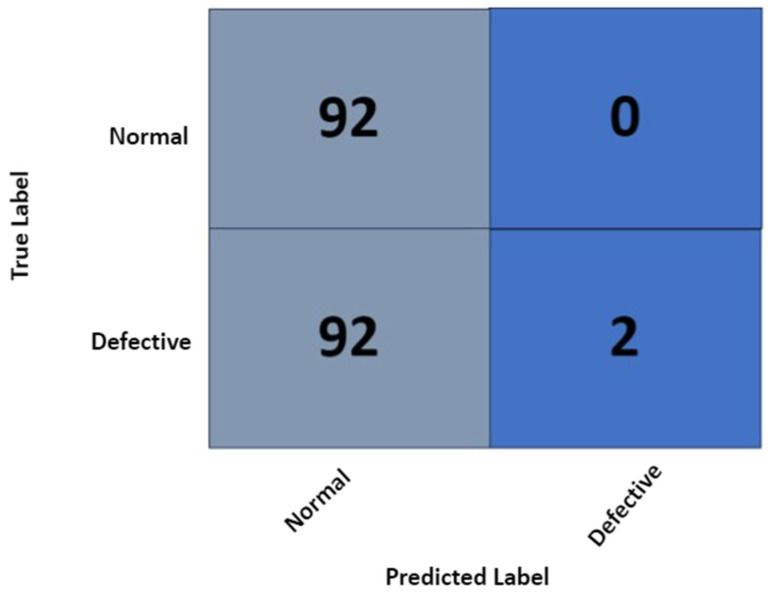
Confusion matrix for the initial model evaluation metrics with original dataset.

Figure 11 complements the training and validation results presented in Figure 10 by presenting the resultant confusion matrix. Based on the class-wise breakdown presented via the confusion matrix in Figure 11, it is clear the architecture had failed to generalize and essentially classified most samples as normal PV cells.

Further breaking down the performance metrics, Table 7 presents the precision, recall, and F1-score for the trained classifier. As evident from the overall F1-score of 67%, it can be concluded that the architecture lacked the generalization capacity with respect to the application.

### 3.3. Augmented Dataset Performance

Based on the performance of the initial architecture, there were two potential routes that could be pursued: firstly, increasing the architectural capacity of the network via increased internal layer depth, and secondly, applying data augmentations. As the objective of the research was to propose a lightweight architecture, the latter option was given priority, as this would not increase the computational complexity of the proposed architecture. Hence, the proposed augmentations based on production floor manifestations (presented in the methodology section) were applied to the initial dataset, and the transformed dataset presented in Table 4 was used for training the initial architecture. 

**Figure 12:**
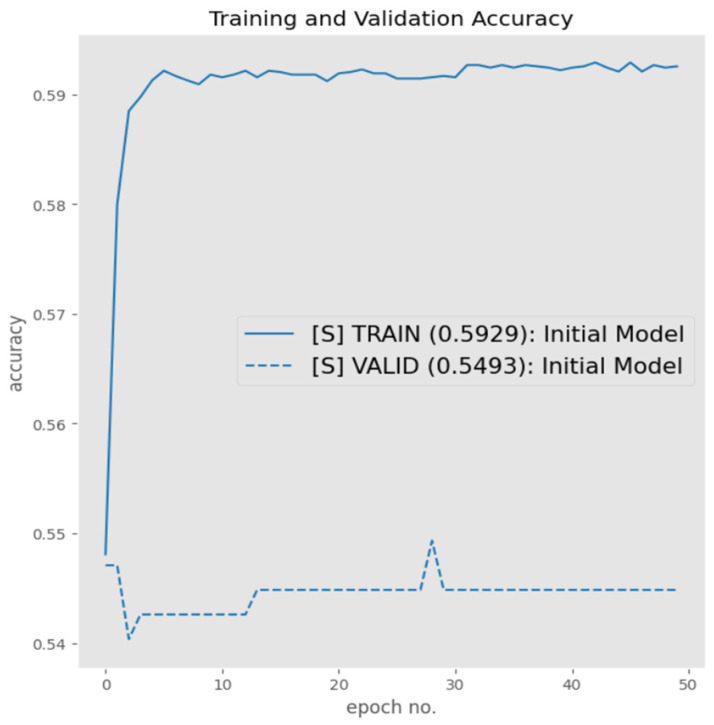
Augmented dataset performance.

Figure 12 represents the performance of the augmented dataset trained on the initial architecture. From Figure 12, it is evident that the augmented dataset did not have any significant impact on improving the performance of the architecture. This did not, however, render the augmentations as ineffective, as mentioned earlier. The reason for the poor performance could be due to the architecture lacking the internal architectural capacity required for generalization on the given dataset. 

### 3.4. Modified Architecture

**Figure 13:**
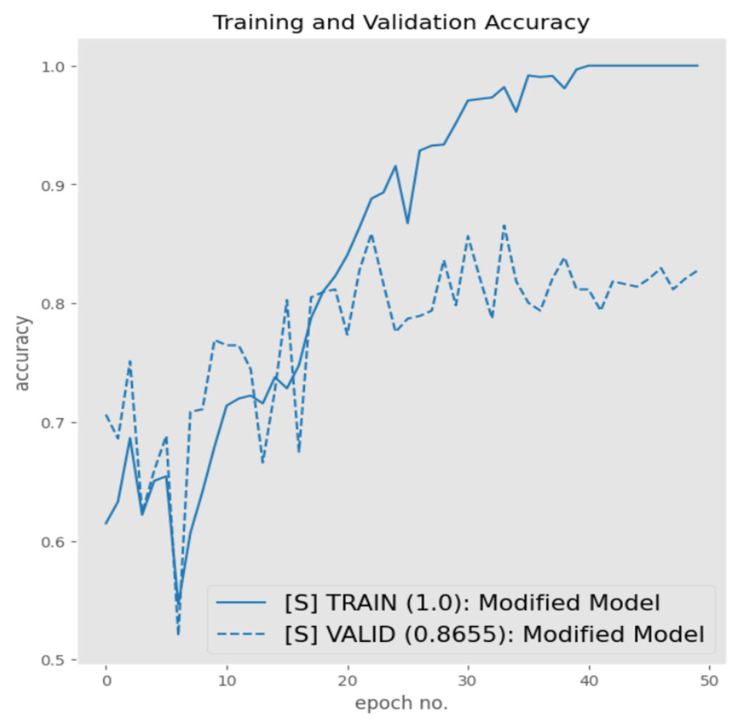
Modified architecture performance.

The next iteration was based on enhancing with internal architectural capacity of the architecture by introducing an additional convolutional block with increased filters and another fully connected layer, the details of which are presented in the Proposed Architecture section. Based on the training/validation graph presented in Figure 13, this iteration had a profound impact on the performance with a validation accuracy reaching 86.6%. The validation curve improved from 54.9% to 86.6%, demonstrating the improved ability of the modified architecture to better generalize because of the introduction of an additional convolutional block. The metric breakdown presented via Table 8 also endorses the performance reported in Figure 13, with an overall F1-score of 84% and improved precision (78%).

#### 3.4.1. Modified Architecture with Batch Normalization

Although the modified architecture provided improved results, when observing the training and validation graphs in Figure 13, it is evident that there is a significant difference between the two respecting accuracies. This indicated that there was a high degree of overfitting being experienced by the trained architecture. Hence, with the aim of reducing overfitting, several regularization strategies were deployed in an iterative manner, starting with batch normalization, with the aim of reducing internal covariance that may be residing with the internal samples of the two classes.

**Figure 14:**
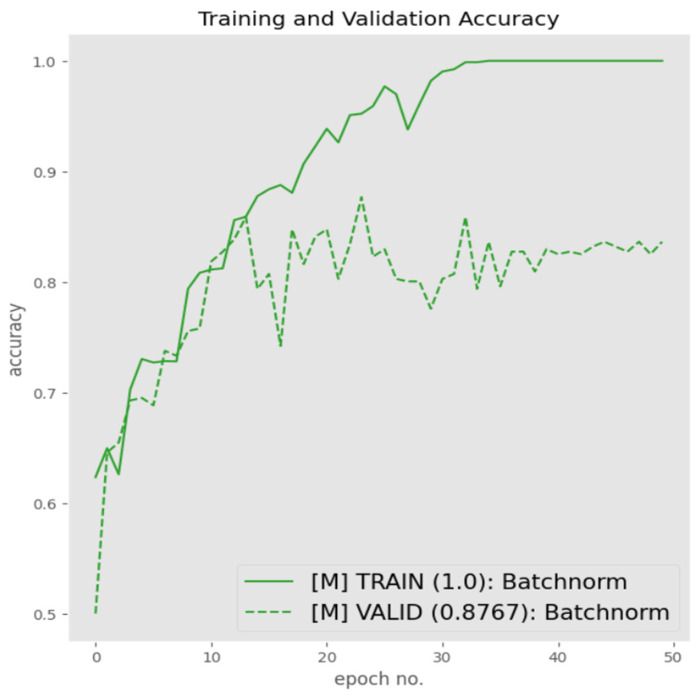
Performance of modified architecture with batch normalization.

Figure 14 presents the performance of the proposed architecture post integration of batch normalization. It is evident from Figure 14 that the introduction of batch normalization did not have any significant impact with respect to reducing overfitting, with the validation accuracy improving by 1%.

#### 3.4.2. Modified Architecture with Dropouts

**Figure 15:**
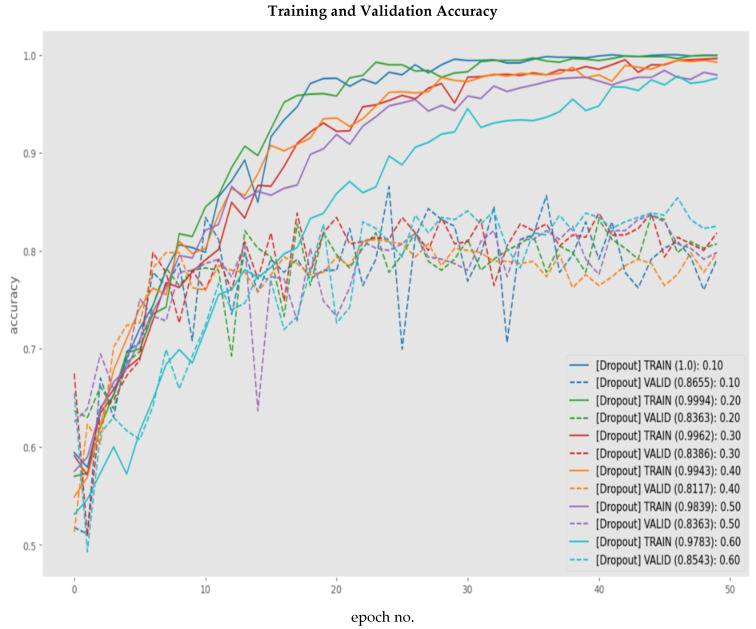
Comparison of modified architecture with dropout rate (10% to 60%).

As the integration of batch normalization did not have a significant impact on reducing overfitting, the next regularization strategy selected was dropout. Dropout would focus on reducing the distance between the training and validation accuracies, also known as reducing the degree of overfitting. The implementation of dropout was based on the drop-ratio parameter, i.e., the ratio of neurons to be randomly disabled. To select the optimal drop-ratio, incremental steps of 10 were taken for the experimentation process, focusing our evaluation on the degree of overfitting. Due to the lightweight architecture of the proposed network, it was important to experiment with different dropout ratios rather than select 0.5 as the default. Figure 15 presents the training and validation graphs for each drop-ratio experiment. As evident from Table 9, a drop-ratio of 0.6, i.e., disabling 60% of the internal neurons of the proposed architecture resulted in the lowest degree of overfitting, i.e., 12.4% with an overall F1-score of 83%.

### 3.5. Modified Architecture with Batch Normalization and Dropout

One final experiment stimulated via intuition was the amalgamation of both batch normalization and dropout into the training and validation pipeline in a synchronous manner with the aim to observe whether this could result in further performance accentuation with respect to reducing overfitting and improving the overall F1-score.

**Figure 16:**
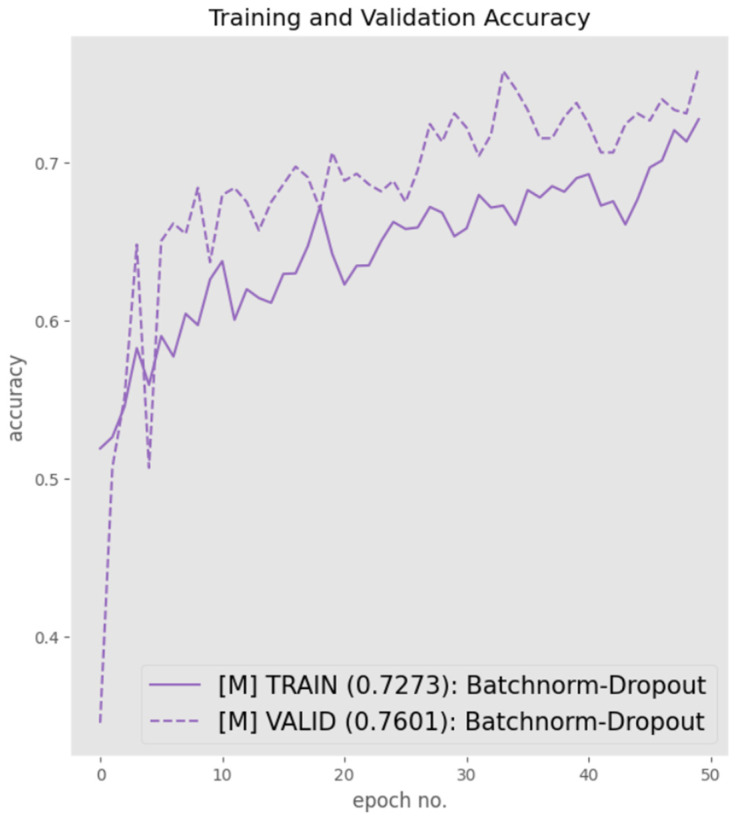
Model performance using a combination of batch normalization and 60% dropout.

This strategy was implemented by integrating batch normalization component and the optimal performing drop-ratio, i.e., 0.6, into the proposed architecture. Figure 16 presents the training and validation graphs. It is evident from Figure 16 that although the degree of overfitting was significantly reduced to less than 5%, the performance with respect to precision, recall, and F1-score had also diminished, with an overall F1-score of 78%, as shown in Table 10.

Based on the result of the dropout, we decided to modify the architecture with a combination of batch-normalization and 60% dropout. Although, the degree of overfitting was minimal compared to the previous histories, the model performance which is represented in Figure 16 and Table 10, respectively, is low when compared to previous analysis in terms of accuracy and F1-Score.

## 4. Discussion

To select the most appropriate architecture configuration for the respective domain, i.e., EL-based PV fault detection, this research presented a development pipeline introducing incremental improvements. Rather than reporting the final proposed solution by itself, we went through the training and validation process after the introduction of each design component to manifest its impact, starting from the original dataset, as evident from Table 11.

A key takeaway from the results presented in Table 11 is that data augmentations and architectural capacity complement each other when it comes to achieving better performance. Although the addition of representative data augmentations improves the variance of the original dataset, it is also necessary to ascertain the basic generalization capacity with respect to the internal architecture, as without enough convolutional blocks and internal filters, the architecture would not be able to extract the key underlying feature characteristics of the dataset in order to provide high performance.

In terms of the final selection, the proposed architecture was selected with the integration of batch normalization, reporting, and an overall F1-score of 85%. Although, one may argue, the proposed architecture with drop-ratio of 0.6 should be selected due to reduced degree of overfitting (12.4%), while looking at the wider application, this may have a negative impact on the architecture post deployment. The reason for this was because the proposed architecture consisted of only two convolutional blocks followed by two fully connected layers, hence further reduction in the network via the application of dropout may reduce the generalization capacity when dealing with wider variance, post deployment.

## 5. Conclusions

In conclusion it can be stated that the research objective, i.e., creating a lightweight architecture for micro-crack detection in PV cells was achieved to a high degree. The lightweight footprint of the architecture is evident from the comparison against state-of-the-art architectures presented in Table 12. It is clear from the comparison, that our proposed architecture was significantly more computationally friendly compared to architectures such as ResNet at 11.69 Million parameters and AlexNet at 61.1 Million parameters. In order to make sure the proposed architecture was able to generalize the PV domain, several augmentations were proposed based on the modeling of a production floor environment. In addition to this, multiple regularization strategies were deployed for obtaining higher convergence. 

As future development, the authors aim to further increase architectural generalization by widening the experimental process to include hyperparameter tuning along with optimizer selection and gradient-based augmentation generation, as presented in [22].

The proposed development pipeline can be extended to similar applications focused on vision-based automated detection implementations requiring limited computational constraints, such as healthcare [23], security [24], food industry [25], renewable energy [26], and other constrained environments [27].

## Figures and Tables

**Table 1:** Original dataset.

Class	Samples
Normal	469
Defect	461

**Table 2:** Original Dataset Split.

	Normal	Defect	Total	Percentage
Testing Set	44	49	93	10%
Training Set	331	320	651	70%
Validation Set	94	92	186	20%
Total	469	461	930	

**Table 3:** Augmented dataset.

Class	Samples
Normal	1131
Defect	1101

**Table 4:** Augmented dataset split.

	Normal	Defective	Total	Percentage
Testing Set	114	109	223	10%
Training Set	791	772	1563	70%
Validation Set	226	220	446	20%
Total	1131	1101	2232	

**Table 5:** Internal depth architecture layout.

Layers	Output Shape	Parameters
Input	3.224 × 224	---
Convo2d-1	8.222 × 222	224
BatchNorm2d	8.222 × 222	16
ReLu	8.222 × 222	---
Max-Pool2d	8.111 × 111	---
Convo2d	16.109 × 109	1168
BatchNorm2d	16.109 × 109	32
ReLu	16.109 × 109	---
Max-Pool2d	16.54 × 54	---
Dropout	16.54 × 54	---
Fc1	100 Neurons	4,665,700
ReLu	100	---
Dropout	100	---
Fc2	50 Neurons	5050
ReLu	50	---
Dropout	50	---
Output	2 Neurons	102
	Total Parameters	4.67 Million

**Table 6:** Hyperparameters.

Global Hyperparamters	
Batch Size	32
Epochs	50
Optimizer	SGD-M
Learning Rate	0.02

**Table 7:** Original dataset performance.

Performance on Original Dataset	
Precision	51%
Recall	100%
F1-Score	67%
Accuracy	50.54%

**Table 8:** Modified architecture performance.

Modified Architecture Performance	
Precision	78%
Recall	91%
F1-Score	84%
Accuracy	86.55%

**Table 9:** Comparison of modified architecture’s performance with dropout.

Dropout Rate	Training Accuracy	Validation Accuracy	Degree of Overfitting	F1-Score
10%	100%	86.55%	13.45%	82%
20%	99.94%	83.63%	16.31%	82%
30%	99.62%	83.86%	15.76%	84%
40%	99.43%	81.17%	18.26%	82%
50%	98.39%	83.63%	14.76%	82%
60%	97.83%	85.43%	12.4%	83%

**Table 10:** Modified architecture with batch normalization and 60% dropout performance.

BN-Dropout Combined Performance	
Precision	73%
Recall	84%
F1-Score	78%
Accuracy	76.01%

**Table 11:** Comparison of model’s performance across all parameters.

Complete Experimental Performance Evaluation	
Original Data Performance	
Precision	51%
Recall	100%
F1-Score	67%
Accuracy	50.54%
Augmented Dataset Performance	
Precision	60%
Recall	19%
F1-Score	28%
Accuracy	54.93%
Modified Architecture Performance	
Precision	78%
Recall	91%
F1-Score	84%
Accuracy	86.55%
Modified Architecture with Batch Normalization Performance	
Precision	79%
Recall	92%
F1-Score	85%
Accuracy	86.67%
Modified Architecture with 60% Dropout Performance	
Precision	81%
Recall	85%
F1-Score	83%
Accuracy	85.43%
Modified Architecture with Batch Normalization and 60% Dropout Performance	
Precision	73%
Recall	84%
F1-Score	78%
Accuracy	76.01%

**Table 12:** Architectural comparison.

Model	Parameters (M)
Proposed	4.7
GoogleNet	13
AlexNet	61.1
ResNet	11.69

## Data Availability

Not presently available.
